# Development of a Diabetes-Focused Print Health Literacy Scale Using the Rapid Estimate of Adult Literacy in Medicine Model

**DOI:** 10.3928/24748307-20201110-01

**Published:** 2020-12-11

**Authors:** Miyong T. Kim, Li Zhushan, Tam H. Nguyen, Nicole Murry, Jisook Ko, Kim B. Kim, Hae-Ra Han

## Abstract

**Background:**

A diabetes mellitus (DM)-specific health literacy (HL) measure that focuses on both oral and print HL is needed in clinical and research settings.

**Objective:**

The present study developed a psychometrically sound DM-specific HL instrument that measures oral and print HL.

**Methods:**

We developed the measure in three steps. First, we reviewed clinical guidelines and conducted focus groups with experts to generate items. Next, we conducted a psychometric evaluation of the scale in three language versions (English, Spanish, and Korean). Lastly, we identified and removed items with potential cultural bias and duplicate functions to produce shorter versions of the scale, using item response theory (IRT).

**Key Results:**

We initially developed an 82-item DM-specific oral HL scale using the Rapid Estimate of Adult Literacy in Medicine (DM-REALM) model. To improve the clinical utility of the DM-REALM, we created shorter forms, a 40-item and 20-item version, and evaluated them by using IRT. All DM-REALM versions had high Cronbach alphas (.985, .974, and .945, respectively) and yielded sufficient convergent validity by positive correlations with existing functional HL scale (*r* = .49, *p* < .001), education (*r* = .14, *p* = .14 to *r* = .54, *p* < .001), and DM knowledge (*r* = .04, *p* = .70 to *r* = .36, *p* < .001). DM-REALM also demonstrated adequate sensitivity as an intervention evaluation tool that captures the changes induced by an intervention.

**Conclusions:**

All forms of the DM-REALM tool were reliable, valid, and clinically useful measures of HL in the context of DM care. Both researchers and clinicians can use this tool to assess DM-specific HL across multiple racial and ethnic populations. **[*HLRP: Health Literacy Research and Practice*. 2020;4(4):e237–e249.]**

**Plain Language Summary:**

This article reported the process and findings of a newly developed health literacy scale for people with diabetes mellitus using three different language versions. Both long and short versions of the scale demonstrated adequate validity and reliability.

The importance of health literacy (HL) in the self-management of chronic illnesses such as diabetes mellitus (DM) is well-documented. For example, insufficient HL has been associated with inadequate information-seeking behaviors, which leads to inadequate DM knowledge and self-care activities (Berkman et al., 2011; Brega et al., 2012; Kim et al., 2004; Vandenbosch et al., 2018). Given that DM is a chronic condition that requires a wide range of self-management skills (Mancuso et al., 2010; Osborn et al., 2010), efforts to improve DM-specific HL present a logical and potentially fruitful intervention strategy.

Nevertheless, relatively few interventions have been developed to improve HL skills to produce desirable clinical outcomes in people with chronic diseases. Moreover, empirical results regarding the direct effects between HL and clinical outcomes of glucose control such as A1C levels have been inconsistent (Berkman et al., 2011; Moss, 2014; Schillinger et al., 2002). These gaps in knowledge may have several plausible explanations, but the lack of sensitive DM-related HL assessment tools and inadequate theoretical frameworks to guide research are potential reasons for these misleading findings. Researchers often use an HL measure that does not specifically focus on DM, which may limit sensitivity to capturing true relationships between HL and clinical outcomes.

HL is a multidimensional construct that consists of print literacy as well as numeracy and functional literacy. In general, a brief assessment that focuses on only one of these dimensions might be helpful in a clinical setting, because its brevity may improve clinical utility. Nevertheless, applying partial assessments of HL in the context of theory testing or intervention evaluation might be insufficient, potentially leading to compromised conclusions and findings with false validity. Similarly, using global HL measures in DM intervention research often fails to capture changes achieved by interventions, because such measures' sensitivity is limited. Currently, the most popular global oral/print HL measures are the Rapid Estimate of Adult Literacy in Medicine (REALM) (Murphy et al., 1993) and the Test of Functional Health Literacy in Adults (TOFHLA) (Parker et al., 1995). Both assess broad and general HL levels rather than disease- or context-specific HL (Battersby et al., 1993; Gordon et al., 2002; Hawthorne & Tomlinson, 1997; Schillinger et al., 2002; Williams et al., 1998; Zaslow et al., 2001). A few functional HL measures relevant for DM management have also been developed and presented in the literature (Chew et al., 2004; Huizinga et al., 2008; Ishikawa & Yano, 2008; Weiss et al., 2005). In particular, the Newest Vital Sign (NVS) (Weiss et al., 2005) is increasingly popular; it has demonstrated high clinical utility because it is brief, focusing on the measurement of patients' ability to apply essential numeracy skills on DM context. Researchers generally agree that such a disease- or context-specific HL tool may be more useful when applied to a group of people managing a chronic illness or condition than would a global HL measure (Nielsen-Bohlman et al., 2004).

To make a comprehensive assessment of HL in the context of DM, there is a current need for a DM-specific HL measure that focuses on both oral and print HL in addition to the well-established functional HL measure. Such a comprehensive assessment of DM-specific HL could be useful in clinical settings and in advancing the theoretical understanding of an important factor that is highly associated with better DM self-management. Therefore, we developed a DM-specific HL instrument that measures print HL, using the REALM as a model. This new DM-specific REALM-like measure (DM-REALM) can be used with currently available DM-specific functional HL measures (e.g., the Short-TOFHLA and the NVS) to provide a more comprehensive assessment of DM-specific HL. Because there are close relationships among HL with education (Kim et al., 2004), information-seeking behaviors (Ishikawa & Yano, 2008), and disease specific knowledge (Nielsen-Bohlman et al., 2004; Vandenbosch et al., 2018), we have examined the relationships among the scores from this new HL scale, education level, and DM knowledge to assess the convergent validity of this new measure.

## Theoretical Framework

Because the intended target population of the tool is adults with diabetes, the theoretical premise of this tool was based on critical tenets of the Process-Knowledge model (Chin et al., 2011)—processing capacity, general knowledge, and specific health knowledge. Our newly developed DM-REALM focuses on both general knowledge and specific self-management knowledge related to DM care. Whereas this tool alone may not cover the in-depth processing capacity as with other functional literacy tools, this DM-specific REALM will be essential in assessing important domains of health literacy among adults facing self-care management challenges of DM.

## Research Design and Methods

We used a three-step instrumentation design, beginning with exploratory construction of an item pool, followed by an intervention and a cross-sectional descriptive study to assess the psychometric properties of the newly constructed scale. Study protocols of each study were reviewed and approved by two Institutional Review Boards: University of Texas at Austin and Johns Hopkins University.

### Construction

To create a comprehensive pool of relevant words for the scale, we used several strategies. First, we searched for relevant literature for current practice guidelines such as those authored by the American Diabetes Association (2019) and the Diabetes Prevention Program Research Group (2002); we also searched for educational materials published from reputable entities such as the National Institute of Diabetes and Digestive and Kidney Diseases. In addition, we used accumulated patient counseling data (Kim et al., 2009; Kim et al., 2015; Kim et al., 2016) to explore the most common and critical terminology that patients with DM would frequently encounter. Finally, we conducted a series of focus groups with expert panels including care providers and DM patients to explore barriers and facilitators related to DM self-management; the professional panel consists of a primary care physician (practice experience more than 25 years), a dietician/nutritionist, a nurse practitioner, community nurse, and diabetic educator. The second panel of patient experts were formulated to obtain patient and family-centered perspectives: two patients (one with less than a 2-year DM diagnosis and one with a more than 10-year history of DM), a family member of the person with DM, a lay health worker, and a nurse researcher.

Each focus group discussed questions about the role of HL or HL-related barriers to adequate self-management of DM. Through this process, we developed an extensive pool of 120 words closely related to understanding and/ or managing DM. Once the list of words was compiled, an expert panel of patients, family members, community health workers, nurses, physicians, and dieticians assessed the content validity of the initial item pool. We used Lynn's (1986) content validity index (CVI) to assess each item's appropriateness. The index uses a 4-point Likert scale, with 1 = *not relevant* and 4 = *very relevant*. Only items rated 3 or 4 on average were retained (CVI > .80). In addition, experts were asked to rate the difficulty level of each word into three categories (1 = *low*, 2 = *medium*, 3 = *high*). Items that were too easy or too difficult based on overall distribution scores were removed. CVI > .80 was used as a measure of agreement among the experts. Our initial version of the DM-REALM comprised of 82 items that assessed a person's ability to pronounce selected words related to understanding and managing DM.

### Initial Structure of the DM-REALM

The DM-REALM was designed to measure oral/print HL using the REALM as a model. Each item in the initial pool was carefully examined to create a valid, reliable scale with high utility for both clinicians and researchers. In particular, the following criteria were used to enhance potential utility of the measures: (1) congruency of the item within the context of current DM care guidelines and practices; (2) clear instruction of administration; (3) principle of parsimony; and (4) discrimination ability of the item to assess how well it could distinguish a low-literacy group from a high-literacy group. The 82 items included in the initial version of the DM-REALM were categorized by their difficulty in three columns: 29 low, 28 medium, and 25 high items. The difficulty level assignment of each word was carried out by a series of research team meetings with consultation from patients, family, and lay health workers.

### Modification of Functional HL

To test the convergent validity of the DM-REALM, we chose a functional HL tool: the four comprehension numeracy items from the NVS (Weiss et al., 2005).

### Translation Process

The initial DM-REALM items were translated from English into both Korean and Spanish using a back translation method (Sperber et al., 1994), which was followed by a committee meeting to assess functional and conceptual equivalence (Beaton et al., 2000). Both conceptual and functional equivalence of different language versions was assessed qualitatively with separate bilingual research teams in Korean and Spanish language groups. Each focus group consisted of 5 to 6 bilingual researchers that held a focus group meeting to examine each word and to discuss if the translation was functionally and conceptually equivalent with the original English version. In addition, each language version of the DM-REALM was assessed for its empirical equivalence through examination of statistical properties. Furthermore, item response theory (IRT) analysis was performed on an aggregated sample pool to identify potential cultural bias using differential item functioning (DIF) (Hambleton et al., 1991). IRT is a modern measurement theory that provides a clear way to examine item and test taker characteristics including potential cultural biases (Nguyen et al., 2014)

### Item Reduction Process

**Figure 1.**
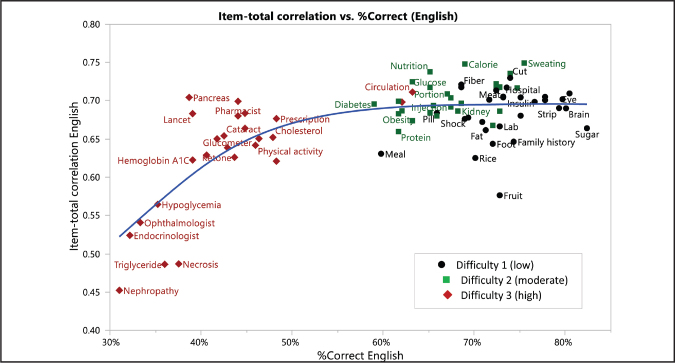
Item-total correlation versus percent correct (English).

Overall, the 82 items showed relatively high Cronbach's alpha (.985 to .988), with item-total correlations ranging from .52 to .83. Because the brevity of a measure is the most important feature in enhancing the clinical utility of a tool, two shorter versions of the measure were considered for further testing, a 40-item and a 20-item scale. Several factors were considered in selecting the items: Cronbach's alpha for different item versions, item-total correlation, balance of items at different difficulty levels, completeness of DM management content domain, and conceptual equivalence of items across the three languages. The conceptual equivalence of each language version was assessed qualitatively through two bilingual DM researchers panel meetings (Korean-English; Spanish-English) to examine if each translated word had the same meaning as the original English word. The reliability assessment was conducted to examine internal consistency of both the 40-item and 20-item versions and found an adequate degree of internal consistency as a whole scale (Cronbach's alpha =.980, and .974 respectively). The item selection process was conducted with the following steps. First, the item-total correlation and the percentage of correct responses were calculated for each item. The item-total correlation measures item discrimination, and percent correct measures item difficulty. Then, the 82 items were placed in an item-total correlation versus percent-correct scatterplot, and a mean curve was fitted with the smoothing splines method (**Figure 1**).

Items with a higher item-total correlation within a percent-correct acceptable range (i.e., the points above the mean curve) were selected. This ensured that the selected items in the shortened scales had the same difficulty distribution as that of the original 82-item scale yet would also have higher discrimination than would the items not selected. Our DIF analysis, described in more detail in the cross-language comparisons using differential item analysis section of this article, revealed significant differences in the way several items functioned across the three languages, so we treated the three linguistic forms for each item separately in calculating the statistics used to select items, and we focused only on the English form in the first round of item selection; after that, in later rounds, we considered the Spanish and Korean forms. Using the English test data, 14 low, 14 medium, and 12 high items were selected for the 40-item version of the scale. From those items, 7 low, 7 medium, and 6 high items were selected for the 20-item version of the scale (**Table 1**).

The ranges of item-total correlations and percent correct for the 82-item original scale, the 40-item scale, and the 20-item scale are summarized in **Table 2**. The item-total correlations for the 82-item scale ranged from .45 to .75, .41 to .84, and .33 to .81 for the English, Spanish, and Korean tests, respectively. After item selection, the 40-item scale had item-total correlation ranges of .52 to .75, .52 to .84, and .42 to .81 for the English, Spanish, and Korean tests, respectively. The 20-item scale had item-total correlation ranges of .52 to .74, .55 to .84, and .42 to .79 for the English, Spanish, and Korean tests, respectively.

### Cross-Language Comparison Using Differential Item Analysis

We fitted a two-parameter logistic IRT model to the English, Korean, and Spanish versions separately. The item difficulty and discrimination parameters of the Korean and Spanish tests were rescaled by equating them to the English test, so that the means and standard deviations of the item difficulty parameters across items were the same for the forms in the three different languages. Comparisons of item difficulty estimates from the Korean and Spanish tests with the English test showed that difficulty shifted for many items, which was confirmed by formal DIF tests. In the English test, there was a distinct separation in difficulty between words in column 3 and words in columns 1 and 2, indicating that the words in column 3 were much more difficult than the words in column 1 and 2 (**Table 1**). In the Spanish and Korean tests, the differences in difficulty between words in column 3 and words in columns 1 and 2 were less distinct. Such differences may be explained by inherent language structure difference among the three languages. Inconsistencies between spelling and pronunciation in English greatly increase the difficulty of correctly pronouncing the more uncommon medical words in column 3, whereas the phoneme-graph-eme correspondence of Spanish and Korean are relatively high, such that words are inherently easier to pronounce.

### Psychometric Properties Assessment

Data from one intervention study (Korean American sample; Kim et al., 2015) and one cross-sectional descriptive study of HL assessments (multiethnic group sample; Murry et al., 2020) were used to test the validity, reliability, and utility of this newly developed DM-REALM scale for HL intervention evaluation.

### Sample

***Study 1 (*n *= 245)***. Research participants in Study 1 were first-generation Korean American immigrants diagnosed with DM and enrolled in a culturally sensitive behavioral education intervention to improve their self-management skills. Participants were recruited from the Korean American community in the greater Washington–Baltimore area. Eligibility criteria for the study were as follows: self-identification as a Korean American immigrant, age 35 years or older; physician-diagnosed DM; difficulty in managing glucose levels, as demonstrated by A1C ≥ 7.0% (53 mmol/mol); and ability to stay in the program for at least 1 year.

***Study 2 (*n *= 389, non-Hispanic White, Hispanic, and Black).*** Participants in Study 2 were recruited from six community clinics within a federally qualified health center (FQHC) that serves patients with low income. Inclusion criteria were as follows: (1) enrolled patients at FQHC, (2) age 18 years or older, (3) medical diagnosis of type 2 DM with a measurement of A1C within the last year, and (4) expressed willingness to participate in all aspects of the study. A total of 404 participants met the eligibility criteria, and 389 completed all questionnaires (261 English-speaking, 128 Spanish-speaking).

### Procedure

After obtaining informed consent, bilingual research assistants (RAs) conducted face-to-face interviews with participants. When we administered the DM-REALM, we gave participants a laminated copy of the DM-REALM in their preferred language and asked participants to pronounce each word. Bilingual RAs scored answers on a laminated copy. If the participant took more than 5 seconds on a certain item, they were told to “skip” the item and move on to the next word. If the participant began to miss every word, the RA would ask the person to pronounce only known words. We marked (−) for any word not attempted or mispronounced. We then counted the number of correct words (+) and recorded the sum score. Informed consent was obtained and all study materials were provided in three language versions (Korean, English, and Spanish).

## Results

### Demographic Characteristics

Demographic characteristics of the samples from the three language groups are summarized in **Table 3**. A total of 634 participants diagnosed with type 2 DM were included in the analysis: 261 English, 128 Spanish, and 245 Korean. The Korean group had a higher percentage of male participants (58.2%) than did the English (36.3%) and Spanish (30.9%) groups. Respondents in the Korean group were older (age 67.6 ± 17.8 years) than those in the two other language groups (English, age 53.6 ± 9.9 years; Spanish, age 51.9 ± 10 years). In the Korean group, the majority were married, 88.8%; in the English group, 25.7% were married; and in the Spanish group, 52.8% were married. The English group reported a slightly higher percentage of having a high school or greater level of education (63.5%) than did those in the Spanish (44.4%) and Korean (52.3%) groups. Participants in both the English and Spanish groups were recruited from a FQHC that provides medical services to primarily patients with low income. Many respondents speaking English or Spanish reported lower than $20,000 for annual income (English, 89.2%; Spanish, 86.9%), whereas only 25.3% of respondents in the Korean group reported annual income below $20,000. Overall, the length of having been diagnosed with DM in the three language groups was 8.8 years (English, 9.8 ± 8.6; Spanish, 8.7 ± 8.4; Korean, 7.9 ± 7.2).

### Reliability Testing

Internal consistency using Cronbach alphas was assessed for the three versions of the DM-REALM (i.e., 82-item, 40-item, and 20-item scales) across the three language groups. The range of Cronbach's alpha for the three language versions of the 82-item scale ranged from .985 to .987. The internal consistency for the two shorter versions was also acceptable, ranging from .945 to .980 (**Table 2**).

### Validity Testing

The face and content validity of the scale were assessed in a series of focus groups, as well as with the use of an expert panel, as described earlier. Based on the rating (1–4) that each expert assigned to items, a CVI was calculated to assess the degree of agreement. Each of the final 82 items achieved a CVI > .80, suggesting strong agreement among the judges and supporting the scale's content validity.

To empirically assess construct validity, we tested the concurrent validity of the DM-REALM with the NVS, a functional HL scale. We defined concurrent validity as demonstrating statistically significant correlations among theoretically related variables; the results are presented in **Table 4**. Specifically, the DM-REALM scale was positively correlated with the NVS (40-item scale, *r* = .46, *p* < .001; 20-item scale, *r* = .45, *p* < .001). We also examined the correlation coefficients between the total scores on the DM-REALM scale and theoretically relevant variables, including education and DM knowledge. DM-REALM, as a new scale, showed positive correlations with both education (*r* = .14, *p* = .14, to *r* = .54, *p* < .001) and DM knowledge (*r* = .04, *p* = .70 to *r* = .36, *p* < .001). The NVS scale was also positively associated with participants' educational level (*r* = .15, *p* < .05 to *r* = .44, *p* < .001) and DM knowledge (*r* = .26, *p* < .05 to *r* = .44, *p* < .001).

## Preliminary Evidence for DM-REALM as an Intervention Evaluation Tool

Since Study 1 was an intervention study, we were able to assess the sensitivity of the DM-REALM as a tool to capture the changes induced by an intervention. Participants in the intervention group demonstrated clear improvements on HL level as measured by both the long and short versions of the DM-REALM. At 12 months postintervention, participants in the intervention group demonstrated better than a 10% improvement on print HL as measured by the DM-REALM (**Table 5**). The changes were much greater during the intervention than in the control group. The differences between the two groups in improvement from baseline were all statistically significant at 6- and 12-months postintervention. Although the level of numeracy, as measured by the NVS also improved, the magnitude of those changes was not as significant and the changes were not as consistent as those measured by the DM-REALM.

## Discussion

The findings of this instrumentation study demonstrate that the DM-REALM has sound psychometric properties. High internal consistency reliability (Cronbach alphas = .985 to .988) was seen across the three language groups. The DM-REALM also yielded sufficient convergent validity by positive correlations with an existing functional HL scale: the NVS (*r* = .49, *p* < .001). Moreover, the DM-REALM scores were statistically significantly associated with theoretically relevant variables such as education (*r* = .53, *p* < .001; *r* = .54, *p* < .01; *r* = .54, *p* < .001) and DM knowledge (*r* = .25, *p* < .01; *r* = .35, *p* < .01; *r* = .36, *p* < .001). Finally, statistically significant changes in DM-REALM scores observed in the Korean sample support the utility of the DM-REALM as a useful intervention evaluation tool. Altogether, these findings indicate that the DM-REALM can be used in clinical settings and for theory testing to elucidate the role of HL in the context of chronic disease management.

The primary motivation for developing the DM-REALM was the recognition of inconsistent findings in the literature regarding the direct relationship between levels of HL and relevant clinical outcomes (Berkman et al., 2011; Moss, 2014; Schillinger et al., 2002). Although it is plausible that HL might influence clinical outcomes indirectly through certain mediators rather than doing so directly, the theoretical understanding of HL cannot progress with insufficient measurement. For example, some researchers have suggested that rather than a direct pathway to clinical outcomes, the role of HL is more prominent through relevant mediators such as information seeking behavior, knowledge, and self-efficacy (Graham et al., 2007; Kalichman et al., 2008; Mancuso, 2010; Mancuso & Rincon, 2006; Osborn et al., 2007; Waite et al., 2008; Wolf et al., 2007). On the other hand, based on null findings, some have argued that HL has a limited role in self-management or in chronic disease management (Bains & Egede, 2011; Kim et al., 2004; Mancuso, 2010; McCleary-Jones, 2011). Incidentally, a separate analysis using the intervention evaluation data obtained from study of the Korean American sample demonstrated that the changes of HL score positively influenced pertinent clinical outcomes such as hemoglobinA1c level (Kim et al., 2020). In the analysis, we used HL as a latent variable comprised of several dimensions of HL including print, functional, and numeracy HL. It was confirmation of our hypothesis that to make more precise inferences about the nature of the relationships between HL and care processes and outcomes, researchers should use a comprehensive HL measurement tool with sufficient sensitivity that directly addresses the context of each study.

Because HL is a multidimensional latent construct that consists of print and numeracy/functional dimensions, omitting the measurement of any one dimension of the construct is not conducive to producing findings with strong inferential validity. The lack of a DM-specific scale for print HL may have contributed to the inconsistent findings or potentially erroneous conclusions of some studies.

## Study Limitations

There are some limitations in this study. The participants in Study 2 were part of a multicultural group, including Hispanic, non-Hispanic White, and Black people. We were able to group Hispanic people by offering a Spanish version of the tool, and the English users were a pool of various racial, ethnic, and cultural groups, including non-Hispanic White and Black people. Therefore, the interpretation of differential item statistics (e.g., DIF) produced by IRT should be limited to differences for different language versions, rather than distinct cultural differences.

Another limitation of our study is associated with our sample characteristics; whereas participants in study 1 were recruited from a community setting, participants in study 2 were recruited from federally qualified community health centers. As a result, they tended to overrepresent people with low levels of education and HL, which results in low variance and subsequently limited statistical power. To improve the generalizability of the DM-REALM scale and set standardized cut-off scores, further studies with participants from a full spectrum of HL levels and various settings are warranted.

The other limitation of this instrumentation study is an inherent interpretation of challenges associated with three different language versions with different degrees of phonetic dominance embedded in their language system. Although uncovering the complex relationships among reading errors and the degree of correspondence between graphemes and phonemes of words in different language systems is beyond the scope of this present research, it is important to understand the potential sources of measurement error of these types of tools as there are systematic differences across languages in the reading processes **(Figures A and B)**. English is known as a language that has relatively deep “orthographic depth” (cf. Katz & Frost, 1992) with low phoneme-grapheme correspondence in comparison to Spanish and Korean. Conversely, both Korean and Spanish languages are based on shallow ortho-graphic depth characterized by high phoneme-grapheme correspondence. Although there is some variability, some argue that testing print HL by pronouncing these selected words might not be a fair assessment strategy of HL as languages with high phoneme-grapheme correspondence allow one to read a word aloud without full understanding of the meaning of the word (Han et al., 2011).

Future HL instrumentation studies should consider these variations of correspondence between graphemes and phonemes of words across different languages and may need to include a test of comprehension in an actual medical context (Nurss et al., 1995; Han et al., 2011).

## Practice Implications

Despite these limitations, the DM-REALM clearly demonstrated its utility as an instrument evaluation tool for DM-specific interventions. As the findings of Study 1 (**Table 5**) indicate, the DM-REALM demonstrated higher sensitivity to capture changes (intervention effects) as compared with a numeracy tool such as the NVS.

Consequently, the DM-REALM produced stronger correlations with theoretically relevant variables such as education and length of stay in the U.S as compared with a global oral/ print scale (the original REALM) and a numeracy HL scale (the NVS). Furthermore, the translated versions of the DM-REALM were subject to rigorous cross-cultural testing, including empirical equivalence testing. Given our conceptual and empirical validation conducted in both the Korean and Hispanic groups, the tool can be used for a variety of DM-specific behavioral intervention programs. Last, the validation of this new instrument supports our theoretical proposition that print HL is an important part of measuring HL comprehensively along with numeracy and other functional HL measures. The findings clearly suggest that measuring functional HL alone is not sufficient for theory-building research or intervention evaluation.

## Conclusion

Researchers and clinicians who use HL as a primary variable or intervention strategy in DM-related research or practice should use a comprehensive measurement approach that includes DM-specific print HL such as the DM-REALM to capture the full dimensions of the latent HL construct. Comprehensively assessing HL in this manner will promote a clearer theoretical understanding of the potential role of HL in chronic disease management. It will also support innovative HL interventions and help propel relevant science forward, ultimately improving the quality of life for people with DM.

## Figures and Tables

**Table 1:** Selected Items for the 40-Item Long Scale and 20-Item Short Scale (English, Spanish, and Korean)

**40-Item Scale**					

**Column 1: Low level of HL**		**Column 2: Medium level of HL**		**Column 3: High level of HL**	

Item 2	Eye	Item 1	Dizzy	Item 2	Circulation
Item 6	Fiber	Item 2	Fatigue	Item 4	Hypoglycemia
Item 8	Meat	Item 8	Fluid	Item 5	Endocrinologist
Item 12	Heart	Item 9	Portion	Item 10	Hyperglycemia
Item 13	Blood	Item 13	Calorie	Item 11	Dialysis
Item 15	Hospital	Item 14	Infection	Item 13	Prescription
Item 16	Vision	Item 15	Stroke	Item 14	Amputation
Item 17	Snack	Item 16	Fasting	Item 15	Pharmacist
Item 19	Insulin	Item 17	Glucose	Item 16	Medication
Item 20	Alcohol	Item 19	Nutrition	Item 17	Lancet
Item 23	Diet	Item 20	Vegetable	Item 18	Pancreas
Item 27	Nerve	Item 22	Swelling	Item 25	Hemoglobin A1C
Item 28	Cut	Item 23	Sweating		
Item 29	Sore	Item 24	Appointment		

**20-Item Scale**					

Item 6	Fiber	Item 2	Fatigue	Item 5	Endocrinologist
Item 8	Meat	Item 8	Fluid	Item 10	Hyperglycemia
Item 13	Blood	Item 9	Portion	Item 11	Dialysis
Item 15	Hospital	Item 15	Stroke	Item 13	Prescription
Item 19	Insulin	Item 17	Glucose	Item 14	Amputation
Item 20	Alcohol	Item 20	Vegetable	Item 25	Hemoglobin A1C
Item 23	Diet	Item 24	Appointment		

**Table 2:** Ranges of Item-Total Correlation and Percent-Correct, and Cronbach Alpha in the Original, Selected Long and Short Scales (*N* = 634)

**Language**	**Psychometric Properties**	**82-Item Original Scale**	**40-Item Long Scale**	**20-Item Short Scale**

English	Median and range for item-total correlation	0.685 (0.45–0.75)	0.705 (0.52–0.75)	0.703 (0.52–0.74)
	Median and range for item percent-correct	67.2% (31%–82%)	68.6% (32%–80%)	68.6 (32%–80%)
	Cronbach alpha	.985	.974	.946

Spanish	Median and range for item-total correlation	0.73 (0.41–0.84)	0.76 (0.52–0.84)	0.775 (0.55–0.84)
	Median and range for item percent-correct	65.2% (37%–80%)	69.1% (41%–77%)	69.9% (41%–77%)
	Cronbach alpha	.988	.974	.945

Korean	Median and range for item-total correlation	0.73 (0.33–0.81)	0.72 (0.42–0.81)	0.73 (0.42–0.79)
	Median and range for item percent-correct	64.1% (8%–87%)	63.5% (14%–87%)	60% (14%–87%)
	Cronbach alpha	.987	.980	.961

**Table 3:** Demographic Characteristics of the Participants

**Characteristic**	**English^a^ (*n* = 261)**	**Spanish^a^ (*n* = 128)**	**Korean^b^ (*n* = 245)**

Sex (%)			
Female	63.7	69.1	41.8
Male	36.3	30.9	58.2

Age (years) ± SD (range, 23–81 years)	53.6 ± 9.9	51.9 ± 10.6	67.6 ± 17.8

Marital status (%)			
Unmarried	74.3	47.2	11.2
Married	25.7	52.8	88.8

Education (%)			
Less than high school	36.5	55.6	47.7
High school or greater	63.5	44.4	52.3

Annual income (%)			
$0–$20,000	89.2	86.9	25.3
$20,001–$39,999	2.2	13	22.4
$40,000–$59,999	0.4		18.5
$60,000–$79,999			11.6
$80,000–$99,999			8.2
$100,000 +			11.6

Length of having diagnosed diabetes mellitus (years) ± SD	9.8 ± 8.6	8.7 ± 8.4	7.9 ± 7.2

Note.

a Collected data from Central Texas.

b Collected data from Central Maryland.

**Table 4:** Construct Validity of the DM-REALM

	**Pearson Product-Moment Correlations (*p* Value)**					

**Language**	**DM-REALM 82-Item**	**DM-REALM 40-Item**	**DM-REALM 20-Item**	**NVS**	**DM Knowledge**	**Education**

DM-REALM 82-item (English)	-	-	-	-	-	-
DM-REALM 40-item (English)	-	-	-	-	-	-
DM-REALM 20-item (English)	-	-	-	-	-	-
NVS	0.04 (.58)	0.12 (.14)	0.15 (.06)	-	-	-
DM knowledge	0.09 (.19)	0.20 (< .05)	0.21 (< .01)	0.44 (< .001)	-	-
Education	0.16 (.08)	0.41 (< .001)	0.47 (< .001)	0.29 (< .01)	0.11 (.33)	-

DM-REALM 82-item (Spanish)	-	-	-	-	-	-
DM-REALM 40-item (Spanish)	-	-	-	-	-	-
DM-REALM 20-item (Spanish)	-	-	-	-	-	-
NVS	0.17 (.07)	0.20 (.07)	0.19 (.08)	-	-	-
DM knowledge	0.03 (.74)	0.04 (.70)	0.05 (.63)	0.26 (< .05)	-	-
Education	0.11 (.44)	0.14 (.47)	0.15 (.43)	0.37 (< .05)	−0.02 (.91)	-

DM-REALM 82-item (Korean)	-			-	-	-
DM-REALM 40-item (Korean)	-	-		-	-	-
DM-REALM 20-item (Korean)	-	-	-	-	-	-
NVS	0.49 (< .001)	0.46 (< .01)	0.45 (< .001)	-	-	-
DM knowledge	0.25 (< .01)	0.35 (< .01)	0.36 (< .001)	0.30 (< .001)	-	-
Education	0.53 (< .001)	0.54 (< .01)	0.54 (< .001)	0.15 (< .05)	0.27 (< .001)	-

Note. DM = diabetes mellitus; DM-REALM = diabetes-specific Rapid Estimate of Adult Literacy in Medicine; NVS = Newest Vital Sign.

**Table 5:** Relative Sensitivity to Capture Changes in Percentages of Correct Responses in Health Literacy by Short and Long Version of DM-REALM (Study1)

**Different Versions of Health Literacy Scales**	**Baseline**	**Month 3**	**Month 6**	**Month 12**

DM-REALM 82-items (0–82), mean % (SE)	62.8 (2.1)	67.9 (2.2)	65.6 (2.4)	67.7 (2.3)
Intervention	65.5 (2.8)	74.4 (2.7)	71.4 (3.1)	74.5 (3)
Control	59.8 (3.2)	60.7 (3.4)	59.2 (3.6)	60.2 (3.5)
Difference	5.7 (4.2)	13.7 (4.3)^**^	12.2 (4.7)^*^	14.3 (4.6)^**^

DM-REALM 40-items (0–40), mean % (SE)	61.8 (2.2)	67.3 (2.3)	65 (2.4)	67.3 (2.4)
Intervention	64.7 (2.9)	73.8 (2.8)	70.8 (3.2)	74.3 (3.1)
Control	58.7 (3.2)	60.2 (3.5)	58.6 (3.7)	59.5 3.6)
Difference	6 (4.3)	13.6 (4.4)^**^	12.2 (4.8)^*^	14.7 (4.7)^**^

DM-REALM 20-items (0–20), mean % (SE)	60.1 (2.2)	65.6 (2.2)	63.9 (2.4)	66.2 (2.3)
Intervention	62.8 (2.8)	71.7 (2.8)	69 (3.2)	73.0 (3)
Control	57.3 (3.2)	58.8 (3.4)	58.2 (3.6)	58.7 (3.5)
Difference	5.4 (4.3)	13 (4.4)^**^	10.9 (4.8)^*^	14.3 (4.6)^**^

NVS (0–6), mean % (SE)	29.3 (2.1)	34 (2.2)	39.5 (2.5)	46.1 (2.7)
Intervention	28.9 (2.8)	39.2 (3.1)	42.7 3.4)	51 (3.8)
Control	29.8 (3.2)	28.2 (3.1)	36 (3.7)	40.7 (3.6)
Difference	0.9 (4.2)	10.9 (4.4)^*^	6.7 (5)	10.3 (5.3)

Note. DM = diabetes mellitus; DM-REALM = diabetes-specific Rapid Estimate of Adult Literacy in Medicine; NVS = Newest Vital Sign; SE = standard error.

* *p* < .05,

** *p* < .01

**Figure A.**
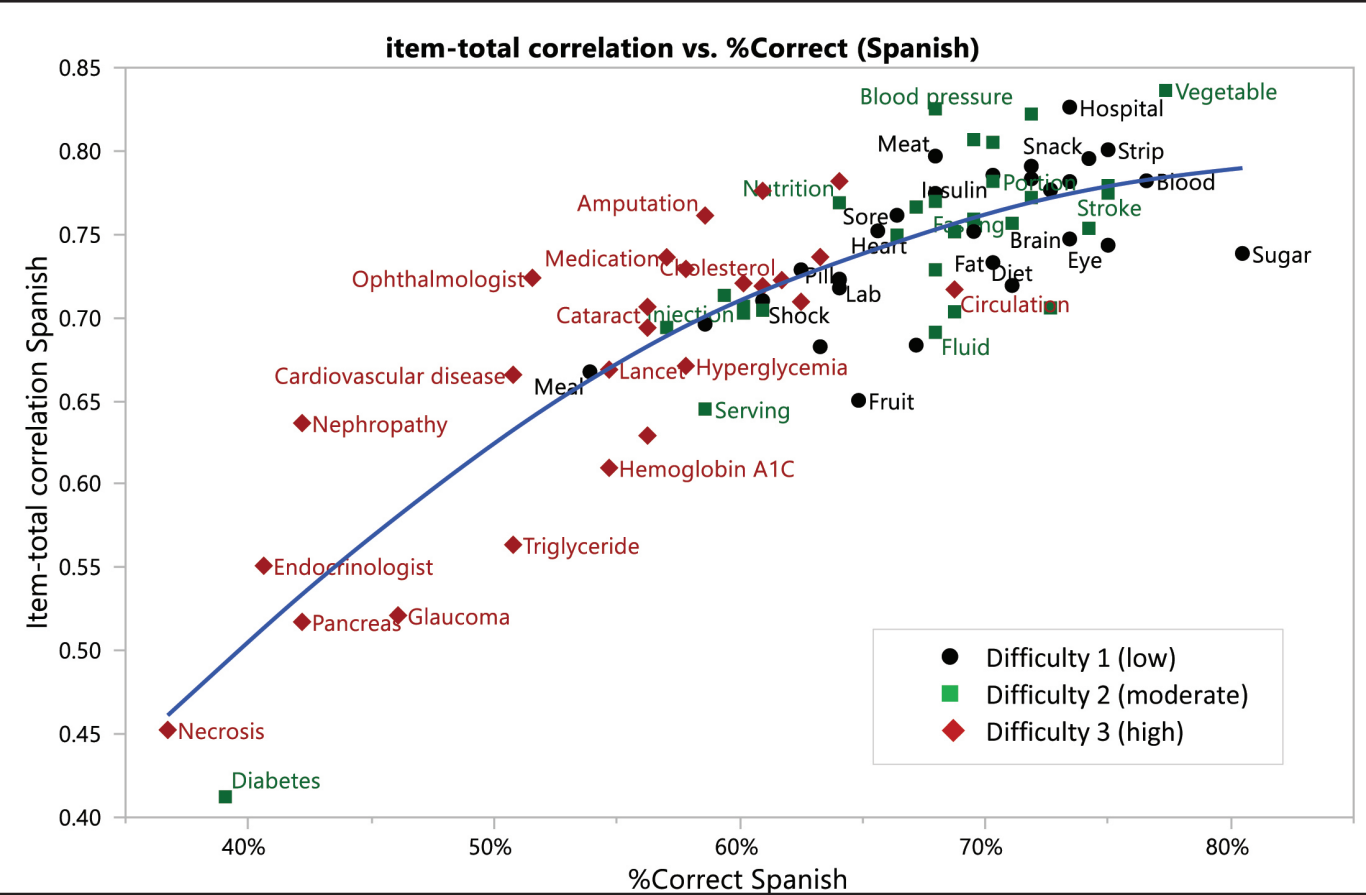
Item-total correlation versus percent correct (Spanish).

**Figure B.**
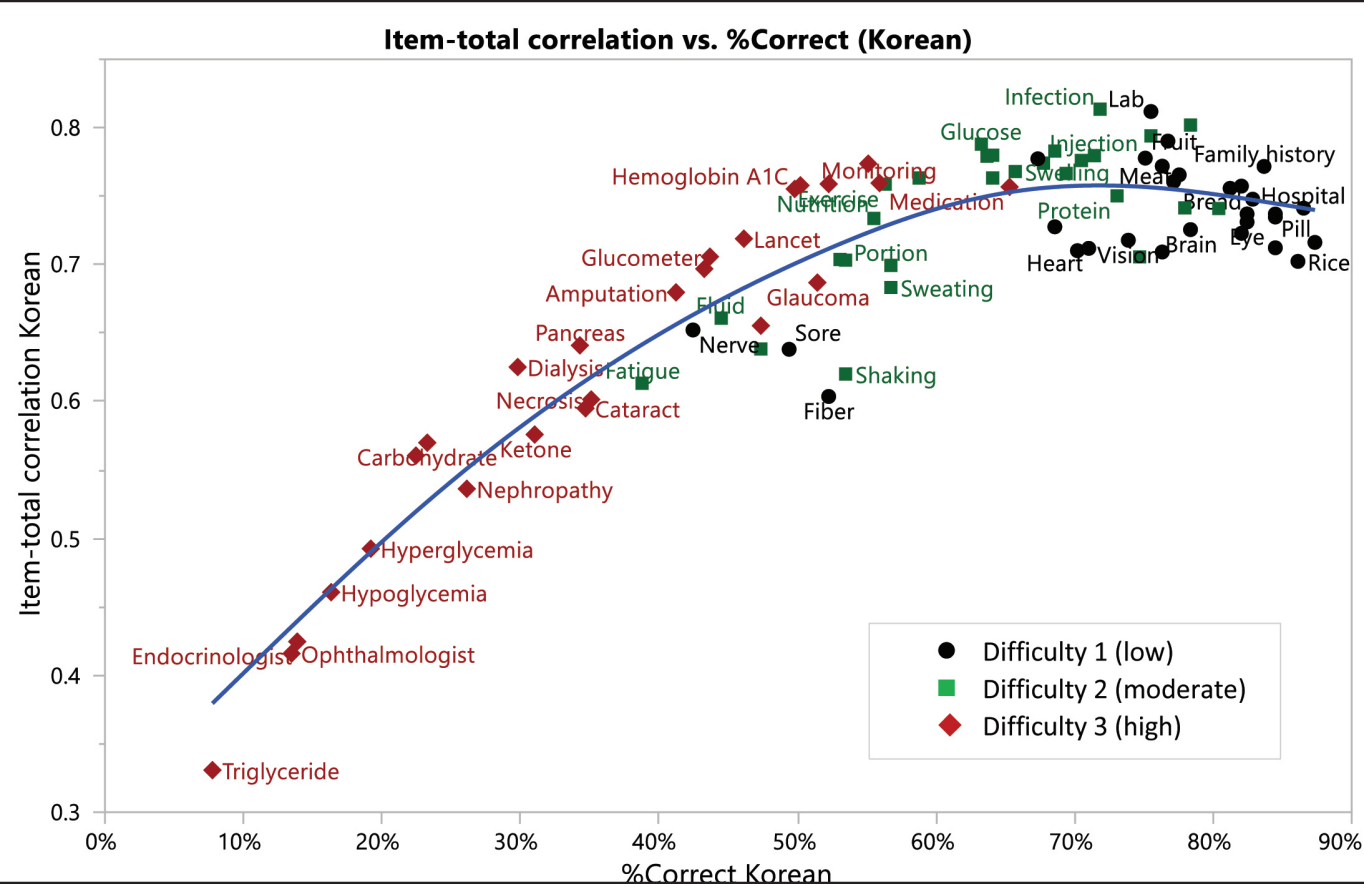
Item-total correlation versus percent correct (Korean).
